# Neck Circumference and Hypertension in Middle-Aged and Older Adults: Baseline Phase Findings of the Ardakan Cohort Study on Aging, Iran

**DOI:** 10.1155/ijhy/6731847

**Published:** 2025-07-22

**Authors:** Mohammad Saatchi, Shadi Naderyan Fe'li, Asma Pourhoseingholi, Mehran Saberian, Mohammad Bidkhori

**Affiliations:** ^1^Department of Biostatistics and Epidemiology, University of Social Welfare and Rehabilitation Sciences, Tehran, Iran; ^2^Health in Emergency and Disaster Research Center, University of Social Welfare and Rehabilitation Sciences, Tehran, Iran; ^3^Department of Epidemiology and Biostatistics, School of Public Health, Tehran University of Medical Sciences, Tehran, Iran; ^4^Iranian Research Center on Aging, University of Social Welfare and Rehabilitation Sciences, Tehran, Iran

**Keywords:** adiposity, aged, anthropometry, hypertension, neck

## Abstract

**Background:** Neck circumference (NC) is a measure to identify upper-body adiposity and has been hypothesized to be linked with hypertension (HTN). This study endeavors to examine the association between NC and HTN among middle-aged and elderly Iranian adults.

**Methods:** In this cross-sectional study, adults over 50 years of age were recruited through a stratified random sampling approach. Anthropometric measurements, blood biochemical indicators, blood pressure (BP) readings, and evaluations of physical activity (PA) levels were conducted.

**Results:** In the univariable regression analyses, age, NC, body mass index, waist and hip circumference, waist-to-hip ratio, total cholesterol, presence of diabetes, PA levels, LDL-C, and HDL-C were found to be associated with HTN in both genders (*p* < 0.2). Notably, triglyceride levels showed a significant association solely among females. Subsequent multivariable regression analyses revealed an association between NC and HTN in both male and female participants (adjusted OR = 1.04 (95% CI: 1.008, 1.08) and 1.06 (95% CI: 1.01, 1.10), respectively).

**Conclusion:** Individuals with higher NC demonstrated an increased likelihood of developing HTN. The strength of this association appeared to be slightly more pronounced in women. Consequently, individuals with larger NC measurements should undergo regular monitoring of BP levels to mitigate potential HTN risks.

## 1. Introduction

Hypertension (HTN) stands as the predominant cause for a myriad of chronic diseases and premature mortality, particularly prevalent in low- to middle-income nations [1]. Due to the high prevalence of elevated blood pressure (BP), a large portion of HTN-related morbidity and mortality is concentrated among the elderly populace. According to the data from the National Health and Nutrition Examination Survey, the prevalence of HTN was noted to be 32% in adults aged 40–59 years and escalated to 70% among the elderly population [2]. Numerous etiologies of HTN have been delineated, with obesity emerging as a pivotal modifiable risk determinant [3, 4]. Despite body mass index (BMI) serving as the conventional anthropometric measure for obesity, its limitation lies in the inability to differentiate between lean muscle mass and fat mass or ascertain body fat distribution [4]. Evidence suggests that adipose tissue centrally located in the body is independently linked to HTN and other cardiometabolic disorders, irrespective of BMI [5]. For that reason, other anthropometric measures such as waist circumference (WC) and waist-to-hip ratio (WHR) have also been used [5]. Neck circumference (NC) is another measure that represents subcutaneous adipose tissue in the neck area and may capture additional risks unaccounted by visceral adipose tissue (i.e., central obesity) [6]. Measuring obesity through NC offers benefits over WC, as it provides accurate results regardless of factors like meals, clothing, or body posture. NC is perceived as a potential indicator for various health conditions and has been associated with components of metabolic syndrome, including central obesity, hypertriglyceridemia, impaired fasting glucose, low serum high-density lipoprotein (HDL-C), and HTN [7, 8].

Previous research has substantiated a link between NC and HTN. A systematic review and meta-analysis demonstrated a pooled odds ratio (OR) of 1.29 (95% CI: 1.06, 1.56) for the relationship between NC and HTN [9]. A Chinese population-based study revealed a statistically significant positive association between NC and systolic blood pressure (SBP) and diastolic blood pressure (DBP) among elderly individuals (linear regression coefficient: 1.054 and 0.382, respectively, *p* < 0.0001) [10]. Moreover, a large population-based study among Iranian elderly cohorts confirmed that NC exhibited a significant association with SBP and DBP levels (mean and standard deviation of NC in subjects with high and normal SBP, respectively, 37.47 ± 3.60 and 36.60 ± 3.59, *p* < 0.001, and mean and standard deviation of NC in subjects with high and normal DBP, respectively, 37.84 ± 3.60 and 36.73 ± 3.59, *p* < 0.001) [11]. Another investigation focusing on Brazilian elderly adults indicated a relationship between larger NC measurements and DBP in both genders (*p* < 0.05), while no statistically significant association was observed in terms of SBP (*p* > 0.05) [12].

The available evidence indicates that the aging trend and prevalence of obesity are rising in the Iranian population [13–15]. On the other hand, a large prospective cohort study has determined the pervasive health issue of HTN within Iran's populace, with an estimated 2.7% developing HTN annually [3]. NC is a relatively novel anthropometric index that specifies adiposity in the upper body region. NC is not typically used in routine healthcare practice in many healthcare settings. In order to use NC as an indicator of HTN risk, it is essential to investigate its association with the condition. Despite certain studies exploring the association between upper body fat aggregation and elevated BP, limited research efforts are geared toward examining this relationship among middle-aged Iranian and older Iranian adults. Hence, the present study aims to determine the association between NC and HTN in the Iranian adult population.

## 2. Methods

### 2.1. Study Design and Setting

This population-based cross-sectional study was performed on participants from the Ardakan Cohort Study on Aging (ACSA), a subset of the Iranian Longitudinal Study on Aging [16], focusing on individuals aged 50 and above in Ardakan County, located in the Yazd province in Central Iran. The ACSA was conducted from 2020 to 2022, utilizing a multistage stratified random sampling approach. Initially, a comprehensive list was compiled comprising all men and women over the aged 50 with active records at all seven healthcare centers. Each healthcare center was considered a distinct stratum. Subsequently, 5819 adults aged 50 years and above were selected through a random sampling process based on the proportional representation of each center in the total number of individuals covered. The study enrolled community-dwelling Iranians aged 50 years and above in Ardakan county who were willing to collaborate with the research team. Criteria for exclusion encompassed individuals with fewer than 2 years of residency in Ardakan County, those with temporary employment status, immigrants, individuals with dementia or severe mental illness, and residents of retirement centers. The protocol of the ACSA was reviewed and approved by the Ethical Committee of the University of Social Welfare and Rehabilitation Sciences, Iran (Ethical code: IR.USWR.REC.1394.490). Data collection procedures ensured confidentiality, and informed consent was obtained from all participants involved in the study.

### 2.2. Measurement and Definition of Study Variables

The age of participants was determined based on the calendar date indicated on their birth certificate. NC was measured at the midcervical spine while participants stood in a straight posture with their arms hanging completely freely by their sides and breathe normally. Participants were weighed by a calibrated scale (seca 755, measurement accuracy of 0.5 kg) wearing light clothes and no shoes. Height was measured in a standing position without shoes, using a fixed measuring tape (seca 206, measurement accuracy of 1 mm), ensuring that the heels, buttocks, shoulder blades, and back of the head were in contact with a wall. In cases where participants were unable to stand unassisted, ulna length, knee height, or demispan were used as alternative measurements of height. Subsequently, the BMI was calculated as the weight in kilograms divided by the height in meters squared. WC was measured between the last rib and the top of the iliac crest using a nonstretchable tape with an approximation of 0.1 cm while participants stood and breathed normally. In addition, hip circumference (HC) was evaluated at the widest portion of the buttocks in a standing position with equal weight distribution on both legs. WHR was calculated by dividing the WC by the HC.

To evaluate the lipid profile and blood glucose levels, blood samples were collected after 8–12 h of fasting. Following centrifugation for 10–15 min at 3000 rpm, serum samples were analyzed using an autoanalyzer. Pars Azmun kit (Pars Azmun Co., Iran) was used to measure blood glucose. Total cholesterol (TC) was measured with a Sarantashkhis kit (Sarantashkhis Co., Iran), and triglyceride (TG) levels were assessed using a Dialab kit (imported by Farsa Med Parsiyan Co., Austria). Low-density lipoprotein (LDL-C) and high-density lipoprotein (HDL-C) were measured using a Biorex kit (Biorex Fars Co., Iran). To determine serum uric acid (SUA), a Pars Azmun kit (Pars Azmun Co., Iran) was also used.

BP was measured in a sitting position using a sphygmomanometer (Omron M6 Comfort, measurement accuracy of 3 mmHg). The measurement was repeated at 10 min intervals, and the average of the two readings was recorded. Participants were advised to avoid vigorous physical activity (PA), consuming coffee or tea, and smoking at least 30 min prior to the BP measurement. HTN was defined as SBP of 140 mmHg or higher and/or a DBP of 90 mmHg or higher, a documented medical history of HTN and/or treatment with antihypertensive medications [17]. Diabetes mellitus (DM) was diagnosed with a fasting blood glucose level of 126 mg/dL or higher, following the American Diabetes Association guidelines or a confirmed medical history [18].

To assess the level of PA, the Physical Activity Scale for the Elderly (PASE) questionnaire was administered. The PASE questionnaire is a commonly used tool to evaluate the frequency and duration of weekly PAs among older adults, encompassing self-reported engagement in occupational, housework, and leisure activities [19, 20]. The questionnaire comprises three main parts: the first part includes six questions concerning leisure time activities, the second part focuses on household activities, and the third part pertains to one question about work-related activities. Scores for each activity are calculated by multiplying the activity's weight by its frequency score, culminating in a total PA score. A higher total score denotes an increased level of PA [21]. The Persian version of PASE indicated acceptable validity and reliability in the study of Hatami et al. [19].

### 2.3. Statistical Analysis

Continuous and categorical variables were presented as the mean ± standard deviation and frequency (percentage), respectively. Unpaired *t*-tests or one-way analysis of variance (ANOVA) were applied to assess differences in the distribution of continuous variables between men and women or NC quintiles. A Chi-square test was also performed to compare categorical variables. Furthermore, both univariable and multivariable logistic regression analyses were conducted to specify the association of NC, functioning as the primary independent variable, and other factors with HTN. Variables demonstrating statistical significance at a level below 0.2 from the univariable logistic regression were subsequently included in the multivariable analysis. In the multivariable analysis, the WHR was categorized as a binary variable to enhance model fit, with distinct cutoff points of 0.8 for women and 0.9 for men. Acknowledging the morphological disparities between males and females, all statistical examinations were conducted separately for each gender. The statistical analyses were executed using STATA_18_ software, with a significance level set at less than 0.05 to ascertain statistical significance.

## 3. Results

The study encompassed a total of 5819 participants, comprising 2939 (50.5%) female individuals and 2880 (49.5%) male individuals, with a mean age of 62.47 ± 7.97 years. The anthropometric and clinical characteristics of the participants, both in aggregate and stratified by gender, are detailed in Table 1.

Almost half of the study participants (50.7%) were found to have HTN. The prevalence of HTN was significantly higher among women compared with men (56.6% vs. 44.8%, *p* < 0.001). Male participants exhibited advanced age and elevated NC, WHR, and PA levels in comparison to their female counterparts (*p* < 0.05). Conversely, BMI, WC, HC, TC, TG, LDL-C, HDL-C, and the prevalence of DM were higher among female participants (*p* < 0.05) (Table 1).

Statistically significant differences were observed in the age of male participants across NC quintiles, displaying a decreasing trend as NC magnitude increased (*p* < 0.001). Conversely, such a trend was not evident among female participants (*p*=0.238). Among both genders, HTN prevalence, BMI, WC, HC, and WHR demonstrated statistically significant differences between quintiles of NC with an increasing trend as the NC increased (*p* < 0.001). While LDL-C levels did not exhibit significant alterations with increasing NC levels in both genders (*p* > 0.05), HDL-C depicted a significant decline with higher NC levels in both genders (*p* < 0.001). No consistent incline or decline pattern with NC was observed in TC and PA levels among men, as well as in TC, TG levels, and DM prevalence among women (Table 2).

**Figure 1:**
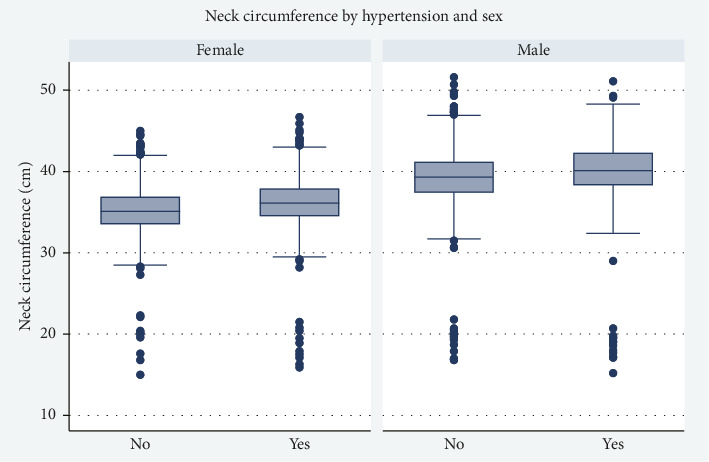
Distribution of neck circumference by hypertension and sex.

The distribution of NC values in relation to HTN is specifically shown in Figure 1.

Following the univariable regression analysis of factors related to HTN, we found that age, NC, BMI, WC, HC, WHR, TC, TG, DM, PA, LDL-C, and HDL-C in men and age, NC, BMI, WC, HC, WHR, TC, TG, DM, PA, LDL-C, and HDL-C in women were eligible to include in a multivariable model (*p* < 0.2) (Table 3). Subsequent adjustment for the effects of all other variables revealed that NC exhibited a statistically significant association with HTN for both male and female participants. Thus, for every one-cm rise in NC, the odds of HTN increased by 4% in men and by 6% in women (adjusted OR = 1.04 and 1.06 for men and women, respectively, *p* < 0.05). It was also observed that there was a statistically significant association between age, BMI, DM, PA, LDL-C, and HTN in men (*p* < 0.05). Moreover, there was a statistically significant association between age, BMI, TG, DM, and HTN in women (*p* < 0.05) (Table 4).

## 4. Discussion

The aim of this study was to investigate the potential association between NC as an indicator of upper-body obesity and the development of HTN among adult participants. The analysis of 5842 individuals highlighted a significant association between NC and HTN in both middle-aged and elderly Iranian men and women, even after adjusting for various risk factors, including age, BMI, WHR, blood lipids, DM, and PA level.

The findings showed that NC was associated with traditional risk factors of HTN, such as general obesity (assessed by BMI), central obesity (measured by WC and WHR), TG, DM, PA level, age, and TC (only in women). In addition, there was a statistically significant association between NC and HDL-C (as a protective factor for HTN). Similar to our study, in a community-based survey including 6431 individuals aged 18–93 years, age, BMI, WC, HC, TC, TG, LDL-C, and fasting blood glucose showed statistically significant differences between NC groups of quintiles (*p* < 0.05), with an increasing trend as the level of NC increased, except for HDL-C which showed a decreasing trend [22]. Moreover, another study demonstrated a statistically significant positive correlation between NC and age, BMI, WC, WHR, TC, TG, LDL-C, and fasting blood glucose in both male and female participants (*p* < 0.05) [23]. Considering the close link between NC and other risk factors, it is possible that they act on the development of HTN through an interactive effect.

Several studies have evaluated the potential value of NC measurement as an indicator of HTN development in adults. For instance, in a survey of 2074 individuals over 65 years old in China, researchers found that NC was positively correlated with both SBP and DBP (SBP: *r*=0.144(*p* < 0.00) in men and *r*=0.214 (*p* < 0.001) in women, and DBP: *r*=0.140 (*p* < 0.001) in men and *r*=0.118 (*p* < 0.001) in women). Moreover, after adjusting for other covariates, the association remained statistically significant for both SBP and DBP and both sexes (*p* < 0.05) [10]. A further cross-sectional study conducted among 1084 Thai adults with a mean age of 54.5 ± 13.1 years showed a statistically significant association between NC and HTN in a multivariable analysis adjusted for covariates of age, DM, dyslipidemia, and BMI among men (adjusted OR = 1.13 (95% CI: 1.04, 1.24), but not statistically significant association for women (adjusted OR = 1.10 (95% CI: 0.01, 1.19) [24]. In addition, a large prospective cohort study demonstrated a statistically significant association between the combination of BMI and NC and the hazard of HTN. Thus, nonobese participants with high NC, obese participants with low NC, and obese participants with high NC had significantly higher hazards of HTN than nonobese participants with low NC, with hazard ratios of 1.066 (95% CI: 1.025, 1.110), 1.322 (95% CI: 1.235, 1.415), and 1.422 (95% CI: 1.337, 1.512), respectively [25]. Moreover, in a cross-sectional study of the Iranian population including 2426 people aged 60 years old and above, the researchers found that the NC measure was significantly associated with both SBP and DBP. In their study, the mean ± standard deviation of NC in participants with high and normal SBP was 37.47 ± 3.60 and 36.60 ± 3.59, respectively (*p* < 0.001). Among individuals with high and normal DBP, the mean ± standard deviation of the NC was 37.84 ± 3.60 and 36.73 ± 3.59, respectively (*p* < 0.001) [11].

These consistent findings across various populations suggest that NC may serve as a practical anthropometric indicator for HTN risk across different ethnic and age groups. However, some variability in the strength of associations, especially between sexes, highlights the need for population-specific NC cutoff points.

Our univariable regression analysis showed a relatively strong and statistically significant relationship between NC and the development of HTN in both sexes. The effect size was almost the same (less than 10% change) and remained statistically significant after adjusting for potential covariates, showing that this association cannot be confounded. These findings are consistent with the previous literature. This consistency may be attributable to the similarity in lifestyle factors across the study populations, the population-based nature of the studies, and the resemblance in adjustment factors of multivariable analyses. Moreover, our multivariable model demonstrated a stronger association between NC and HTN among women than men participants (adjusted OR and 95% CI = 1.06 (1.01, 1.10) vs. 1.04 (1.008, 1.08)). Concordant with this observation, an analysis of data from the Framingham Heart Study demonstrated that, after controlling covariates, NC had a stronger association with HTN in women than in men [26]. The difference in the way of storing free fatty acids in the subcutaneous tissues of men and women may be an explanation for this difference [27, 28]. Moreover, the slightly stronger association between NC and HTN in women may reflect underlying differences in fat distribution and hormonal influences. Women typically have a higher percentage of subcutaneous fat, including in the upper body, and NC may more accurately reflect central adiposity in women compared with men. This regional adiposity, particularly in the upper body, has been linked to cardiometabolic risk factors, including elevated BP. In addition, hormonal factors such as estrogen levels, which influence fat distribution and endothelial function, may modulate the relationship between NC and HTN in women. Postmenopausal hormonal changes, for example, are associated with increased visceral and upper-body fat deposition, potentially enhancing the relevance of NC as a marker of cardiovascular risk in this population.

Obesity is known to precipitate metabolic complications including insulin resistance, dyslipidemia, and HTN [29]. However, regional fat deposition, especially in the upper limb regions, may be a better indicator of HTN development. Multiple potential mechanisms have been discovered for rising BP associated with NC. It has been found that basal and postprandial free fatty acid increases in obese patients, and subsequently, elevated levels of circulating free fatty acid increase oxidative stress markers and inflammation through the activation of inflammatory pathways and cytokine release. These pathophysiological cascades culminate in vascular endothelial dysfunction, thereby potentially contributing to the development of HTN [30, 31]. Furthermore, increased upper-body adiposity is associated with insulin resistance, which may exacerbate sympathetic nervous system activity and renal sodium retention—both of which contribute to the pathogenesis of HTN [32].

The key strengths of this study include the use of a population-based design, utilizing a random sampling strategy and a considerable sample size to ensure the generalizability of results. Furthermore, the investigators conducted an adjusted analysis to ensure appropriate control of confounding variables. However, several limitations merit consideration when interpreting the results. The cross-sectional design precludes the establishment of a causal relationship between NC and HTN. In addition, the reliance on self-reported PA may introduce measurement error owing to potential lapses in participant recall. Moreover, some possible confounders, such as dietary habits, sleep apnea, and medications use, were not controlled in this study.

## 5. Conclusion

According to the findings of this study, individuals aged over 50 with higher NC measurements were shown to have a greater likelihood of developing HTN. Importantly, this association appeared to be somewhat more pronounced in women compared with men. Considering that NC measurement is a noninvasive, easy, and low-cost method, the findings of this study can be important. Traditional methods of measuring body fat distribution, such as WC and WHR, can be unreliable and constraining methods due to dependence on meals, clothing, breathing, cultural, and social factors. Furthermore, in elderly individuals, various comorbidities such as chronic pain, arthritis, frailty, osteoporosis, and sarcopenia can pose challenges for accurate measurements using these conventional indices, especially when considering factors like the ability to stand upright. In contrast, NC measurement offers a practical alternative that overcomes many of these limitations, making it a viable option for use in both clinical and primary healthcare settings. It is essential to acknowledge that individuals with conditions such as goiter or cervical spine abnormalities may present challenges in accurately measuring NC. In such cases, alternative methods of assessing cardiovascular risk should be considered. In light of these study findings, it is recommended that individuals with larger NC measurements pay attention to monitoring their BP regularly and implementing appropriate preventive measures. This proactive approach could help in identifying and managing HTN risk in a timely manner, ultimately contributing to better cardiovascular health outcomes in at-risk populations, particularly those over the age of 50.

## Figures and Tables

**Table 1:** General characteristics of the participants based on gender.

	Total (*n* = 5819)	Male (*n* = 2880)	Female (*n* = 2939)	*p* value^∗^
Age (years)	62.47 ± 7.97	63.24 ± 8.25	61.71 ± 7.60	< 0.001
NC (cm)	37.66 ± 3.83	39.61 ± 3.64	35.75 ± 2.93	< 0.001
BMI (kg/m^2^)	28.54 ± 4.89	27.03 ± 4.31	29.97 ± 4.97	< 0.001
WC (cm)	99.93 ± 11.16	99.48 ± 11.75	100.38 ± 10.52	0.004
HC (cm)	103.40 ± 9.53	100.81 ± 7.57	105.99 ± 10.54	< 0.001
WHR	0.96 ± 0.07	0.98 ± 0.07	0.94 ± 0.07	< 0.001
TC (mg/dL)	183.41 ± 41.64	176.78 ± 40.88	189.92 ± 41.36	< 0.001
TG (mg/dL)	167.12 ± 95.59	164.60 ± 102.74	169.59 ± 87.95	0.046
LDL-C (mg/dL)	108.85 ± 32.05	106.00 ± 31.70	111.67 ± 33.04	< 0.001
HDL-C (mg/dL)	48.92 ± 10.52	47.72 ± 9.74	52.07 ± 10.31	< 0.001
PA	139.34 ± 86.69	150.58 ± 103.12	128.33 ± 64.93	< 0.001
HTN	2955 (50.78)	1290 (44.80)	1665 (56.65)	< 0.001
DM	2287 (39.30)	1024 (35.56)	1263 (42.97)	< 0.001

*Note:* Data are expressed as the mean ± standard deviation. TG: triglyceride, HTN: hypertension.

Abbreviations: BMI, body mass index; DM, diabetes mellitus; HC, hip circumference; HDL-C, high-density lipoprotein cholesterol; LDL-C, low-density lipoprotein cholesterol; NC, neck circumference; PA, physical activity; SUA, serum uric acid; TC, total cholesterol; WC, waist circumference; WHR, waist-to-hip ratio.

^∗^Independent sample *t*-test.

**Table 2:** Characteristics of participants by neck circumference quintiles.

	Sex	Quintiles of neck circumference (cm)					*p* value^∗^
		Q1	Q2	Q3	Q4	Q5	
Age (years)	Male	66.29 ± 9.29	63.92 ± 8.52	63.54 ± 8.35	63.12 ± 8.31	62.56 ± 7.80	< 0.001
	Female	61.60 ± 7.77	61.60 ± 7.54	62.11 ± 7.46	62.26 ± 7.39	62.81 ± 7.75	0.238

BMI (kg/m^2^)	Male	20.62 ± 4.13	22.50 ± 2.53	24.48 ± 2.45	26.87 ± 2.65	30.49 ± 3.52	< 0.001
	Female	26.43 ± 3.77	30.04 ± 3.54	32.55 ± 3.99	34.69 ± 3.83	37.89 ± 5.09	< 0.001

WC (cm)	Male	82.53 ± 13.52	87.71 ± 9.63	93.48 ± 7.09	99.36 ± 7.46	108.49 ± 8.97	< 0.001
	Female	93.24 ± 9.03	100.60 ± 7.89	105.67 ± 7.79	109.41 ± 7.97	116.46 ± 8.54	< 0.001

HC (cm)	Male	91.08 ± 7.34	94.15 ± 4.62	97.05 ± 4.63	100.58 ± 5.27	106.30 ± 6.98	< 0.001
	Female	100.14 ± 8.64	105.97 ± 8.58	110.56 ± 9.78	114.17 ± 9.71	119.07 ± 11.28	< 0.001

WHR	Male	0.89 ± 0.09	0.93 ± 0.08	0.96 ± 0.05	0.98 ± 0.05	1.02 ± 0.06	< 0.001
	Female	0.93 ± 0.07	0.94 ± 0.06	0.95 ± 0.06	0.95 ± 0.07	0.97 ± 0.07	< 0.001

TC (mg/dL)	Male	179.94 ± 36.21	178.04 ± 37.91	175.82 ± 38.60	175.86 ± 41.93	177.25 ± 42.61	0.740
	Female	192.89 ± 41.01	191.71 ± 40.74	184.81 ± 41.17	187.04 ± 40.94	184.12 ± 48.05	< 0.001

TG (mg/dL)	Male	112.48 ± 62.45	129.81 ± 79.32	147.47 ± 84.66	165.82 ± 99.58	190.0 ± 116.36	< 0.001
	Female	151.81 ± 82.56	176.56 ± 95.06	173 ± 80.63	193.44 ± 87.52	193.10 ± 88.52	< 0.001

HTN	Male	41 (29.93)	98 (31.41)	165 (39.96)	381 (44.88)	551 (53.29)	< 0.001
	Female	472 (45.17)	511 (58.60)	398 (65.68)	206 (67.99)	78 (69.039)	< 0.001

DM	Male	26 (18.98)	66 (21.15)	165 (30.11)	22 (33.22)	485 (46.91)	< 0.001
	Female	332 (31.77)	371 (42.55)	321 (52.97)	156 (51.49)	83 (73.45)	< 0.001

PA	Male	150.21 ± 104.48	168.45 ± 104.72	159.34 ± 103.09	152.17 ± 102.40	139.36 ± 101.94	< 0.001
	Female	134.28 ± 67.33	130.48 ± 62.77	124.00 ± 64.37	120.70 ± 62.16	100.71 ± 58.85	< 0.001

LDL-C (mg/dL)	Male	105.98 ± 28.10	106.35 ± 30.97	104.67 ± 30.33	106.63 ± 32.43	106.13 ± 34.49	0.871
	Female	112.52 ± 32.56	112.86 ± 32.73	109.42 ± 32.90	111.40 ± 33.70	108.23 ± 37.69	0.249

HDL-C (mg/dL)	Male	52.70 ± 10.0	49.27 ± 10.47	47.47 ± 9.66	44.92 ± 9.31	43.43 ± 8.93	< 0.001
	Female	54.74 ± 10.67	51.62 ± 9.66	50.31 ± 9.61	49.58 ± 10.63	47.80 ± 8.84	< 0.001

*Note:* Data are expressed as the mean ± standard deviation or *n* (%). TG: triglyceride, HTN: hypertension.

Abbreviations: BMI, body mass index; DM, diabetes mellitus; HC, hip circumference; HDL-C, high-density lipoprotein cholesterol; LDL-C, low-density lipoprotein cholesterol; PA, physical activity; TC, total cholesterol; WC, waist circumference; WHR, waist-to-hip ratio.

^∗^Independent sample *t*-test or ANOVA.

**Table 3:** Univariable logistic regression analysis of neck circumference as the main independent variable and other factors related to hypertension, stratified by sex.

	Men		Women	
	Crude OR (95% CI)	*p* value	Crude OR (95% CI)	*p* value
Age (years)	1.06 (1.05, 1.07)	< 0.001	1.07 (1.06, 1.08)	< 0.001
NC (cm)	1.08 (1.05, 1.08)	< 0.001	1.12 (1.09, 1.15)	< 0.001
BMI (kg/m^2^)	1.08 (1.05, 1.09)	< 0.001	1.05 (1.04, 1.07)	< 0.001
WC (cm)	1.03 (1.02, 1.04)	< 0.001	1.03 (1.03, 1.04)	< 0.001
HC (cm)	1.03 (1.02, 1.04)	< 0.001	1.01 (1.00, 1.02)	< 0.001
WHR	2.48 (1.84, 3.33)	< 0.001	1.84 (1.40, 2.42)	< 0.001
TC (mg/dL)	0.99 (0.98, 0.99)	< 0.001	0.99 (0.98, 0.99)	< 0.001
TG (mg/dL)	0.99 (0.99, 1.00)	0.794	1.002 (1.001, 1.003)	< 0.001
DM	2.77 (2.37, 3.24)	< 0.001	3.08 (2.64, 3.560)	< 0.001
PA	0.99 (0.995, 0.996)	< 0.001	0.99 (0.994, 0.996)	< 0.001
LDL-C (mg/dL)	0.98 (0.98, 0.99)	< 0.001	0.98 (0.98, 0.99)	< 0.001
HDL-C (mg/dL)	0.97 (0.97, 0.98)	< 0.001	0.97 (0.96, 0.98)	< 0.001

*Note:* TG: triglyceride.

Abbreviations: BMI, body mass index; DM, diabetes mellitus; HC, hip circumference; HDL-C, high-density lipoprotein cholesterol; LDL-C, low-density lipoprotein cholesterol; NC, neck circumference; OR, odds ratio; PA, physical activity; TC, total cholesterol; WC, waist circumference; WHR, waist-to-hip ratio.

**Table 4:** Multivariable logistic regression analysis of the association between neck circumference and hypertension adjusted for other variables, stratified by sex.

	Men		Women	
	Adjusted OR (95% CI)	*p* value	Adjusted OR (95% CI)	*p* value
Age (years)	1.07 (1.05, 1.08)	< 0.001	1.07 (1.06, 1.09)	< 0.001
NC (cm)	1.04 (1.008, 1.08)	0.016	1.06 (1.01, 1.10)	0.006
BMI (kg/m^2^)	1.05 (1.02, 1.08)	0.001	1.04 (1.01, 1.06)	0.001
WHR	1.27 (0.86, 1.86)	0.223	1.12 (0.80, 1.57)	0.479
TC (mg/dL)	0.99 (0.99, 1.005)	0.800	0.99 (0.98, 1.00)	0.237
TG (mg/dL)	—	—	1.002 (1.00, 1.003)	0.005
DM	1.88 (1.54, 2.29)	< 0.001	2.02 (1.67, 2.45)	< 0.001
PA	0.99 (0.996, 0.998)	< 0.001	0.99 (0.99, 1.00)	0.273
LDL-C (mg/dL)	0.99 (0.983, 0.998)	0.026	0.99 (0.98, 1.00)	0.259
HDL-C (mg/dL)	1.00 (0.989, 1.011)	0.924	0.99 (0.98, 1.01)	0.826

*Note:* TG: Triglyceride.

Abbreviations: BMI, body mass index; DM, diabetes mellitus; LDL-C, low-density lipoprotein cholesterol; NC, neck circumference; OR, odds ratio; PA, physical activity; TC, total cholesterol; WHR, waist-to-hip ratio.

## Data Availability

The data that support the findings of this study are available from the corresponding author upon reasonable request.
